# Examining a Complex Model Linking Maternal Reflective Functioning, Maternal Meta-Emotion Philosophies, and Child Emotion Regulation

**DOI:** 10.3390/children10071161

**Published:** 2023-07-02

**Authors:** Rong Shao, Sitong Liu, Robert J. Coplan, Xi Chen, Junsheng Liu

**Affiliations:** 1 Shanghai Key Laboratory of Mental Health and Psychological Crisis Intervention, School of Psychology and Cognitive Science, East of China Normal University, Shanghai 200050, China; rongshaoaubrey@gmail.com (R.S.); m18321563536@163.com (S.L.); jsliu@psy.ecnu.edu.cn (J.L.); 2 Department of Psychology, Carleton University, Ottawa, ON K1S 5B6, Canada; robert.coplan@carleton.ca; 3 Shanghai Changning Mental Health Center, Shanghai 200335, China

**Keywords:** parental reflective functioning, emotion coaching, emotion dismissing, emotion regulation, mothers

## Abstract

Parental Reflective Functioning (PRF) refers to parents’ ability to understand their children’s behavior in light of underlying mental states such as thoughts, desires, and intentions. This study aimed to investigate whether maternal meta-emotion philosophies (i.e., emotion coaching, emotion dismissing) mediated the relation between maternal RF and child emotion regulation (ER). Additionally, children’s genders and ages were examined as moderators of the associations between maternal RF and maternal meta-emotion philosophies. The sample comprises 667 Chinese mothers of children aged 4–6 years. Mothers completed questionnaires assessing their reflective functioning, emotion coaching and dismissing, and child emotion regulation. Results indicated both a direct link between maternal RF and child emotion regulation, as well as indirect pathways mediated by emotion coaching and dismissing. A child’s gender and age also moderated the relations between maternal RF and meta-emotion philosophies. Specifically, the negative association between maternal pre-mentalizing modes and emotion coaching was stronger for mothers of girls than boys; whereas the negative association between maternal certainty of mental states and emotion dismissing, as well as the positive association between maternal interest and curiosity and emotion coaching were both stronger for mothers of younger children than older children. The findings suggest that emotion coaching and dismissing mediate the relation between maternal PRF and the emotion regulation of children.

## 1. Introduction

Children’s *emotion regulation* (ER) refers to the ability to monitor, evaluate and adjust emotional responses aimed to achieve personal goals and to adapt to social environments [1,2,3]. Children with higher ER abilities are better able to modulate their emotional expression and arousal according to their needs and adopt different strategies to regulate their emotions, which helps them cope with changes in the external social environment [4,5]. Thus, it is important to examine the factors that facilitate the development of children’s emotion regulation abilities. 

The *reflective function* is the ability to understand external behaviors based on underlying mental states and intentions [6]. In interpersonal interactions, individuals with this ability can understand their own and others’ behaviors according to their internal mental states, such as feelings, thoughts, intentions, motivations, or beliefs [7]. Drawing upon the attachment theory, it has been postulated that the *parental reflective function* (PRF) can serve a critical function in children’s emotional development [8]. In the past 10 years, a growing body of empirical research has found that maternal PRF positively predicts both parenting practices and children’s social-emotional development [9,10,11,12,13,14,15,16]. From the perspective of the social information processing model, PRF skill facilitates the cognitive processes with which parents can interpret their own and their children’s intentions and further helps parents to select adaptive responses during mother-child interactions [17]. In this regard, in the current study, we speculated that PRF would promote the emotional development of children by increasing supportive parenting practices toward children’s emotions.

Specifically, parental coping strategies in response to children’s negative emotions have been identified as important factors affecting the development of young children’s emotion regulation [3,18,19]. In particular, parental meta-emotion philosophy (MEP) appears to play a critical role [20,21]. MEP comprises parental beliefs and responses toward their children’s emotions and can be divided into *emotion coaching* and *emotion dismissing* socialization styles [22,23]. Notwithstanding, to what extent emotion coaching and dismissing acted as mediators between PRF and children’s emotional regulation has not been examined in empirical studies. 

Finally, few studies have examined potential moderators of the relations between maternal PRF and children’s emotional development. According to Sharp and Fonagy [24], parents’ capacity to engage in proper mentalizing is influenced not just by their own PRF but also by their children’s characteristics. Accordingly, in the present study, we also explored the moderating effects of a child’s gender and age on the associations between PRF and emotion coaching and dismissing. 

### 1.1. PRF and Children’s Emotion Regulation

The reflective function was introduced by Fonagy and refers to the ability to understand behavior in light of underlying mental states and intentions [6]. This adaptive skill helps individuals better understand themselves and others, making the behavior of people more predictable in interpersonal interactions, and facilitating the formation of social relationships [9,25]. Fonagy and Target [26,27] introduced the concept of parental reflective functioning (PRF) and developed a measure of this construct using the Adult Attachment Interview [28], which considers the ability of parents to reflect on their own and their children’s internal mental states. 

Parents with high PRF are able to relate their own and their children’s mental states to behaviors, and they can not only recognize the internal states, but also make correct attributions of behaviors according to internal states, which can support parents’ desire to understand their children’s inside world [8,29]. Several cross-sectional studies have explored the relation between PRF and other aspects of parenting. For example, positive correlations have been reported between PRF and both parental empathy and emotion regulation [14]. Maternal PRF also positively predicted maternal support during mother-child play [15] and parent-child relationship quality (including parental involvement, communication, satisfaction, and support) [30]. All of these findings suggest the link between PRF and supportive parenting behaviors. 

Along with positive effects on parenting, parents’ abilities to understand their children’s behaviors, by considering internal mental states, also foster the social-emotional development and adjustment of children [13,16,31]. In terms of the underlying mechanisms that may link PRF and children’s emotional outcomes, there is some evidence suggesting that parental sensitivity mediates the relation between PRF and children’s attachment [32,33,34]. Mothers with higher levels of PRF were also found to have more progressive parenting beliefs, which in turn predicted their children’s better emotion understanding [11]. The quality of triadic interactions among mothers, fathers, and children was also found to mediate the relation between maternal PRF and children’s social-emotional difficulties [16]. Finally, parenting competence mediated the relation between maternal PRF and indices of infants’ social-emotional development [13]. These studies suggest parenting and parents’ performance during child-parent interaction as mechanisms linking the relation between PRF and children’s socio-emotional development. Yet, few empirical studies have specifically examined how PRF might affect children’s emotion regulation. 

Emotion regulation is a key contributor to children’s emotional competence, promotes adaptive cognitive and social development [35,36], and reduces the risk of psychopathology [18]. Parenting style, parents’ coping strategies in response to negative emotions, and the quality of the parent-child relationship have been identified as important factors affecting the development of young children’s emotion regulation [3,18,19]. However, less attention has been paid to the potential role of PRF. Indeed, to date, only one study has explored whether maternal PRF moderated the association between children’s observed distress and coping during a challenging task (an index of emotion regulation) [14]. The study found that one dimension of PRF (certainty of mental states) moderated the association between distress and coping. Such that, only for mothers with high certainty of mental states, children’s higher distress was associated with more aggressive coping. This finding provides preliminary support for the positive link between PRF and children’s emotion regulation abilities.

Despite the limited previous empirical research, drawing upon conceptual links between PRF and children’s emotional development [8], it can be postulated that parents with a better mentalizing capacity would encourage their children to be more willing to seek parental support when experiencing negative emotions (e.g., frustration, pain, or sadness). In turn, the positive interactions that occur in this parent-child relationship enable children to internalize emotion regulation strategies more effectively and further promote coping strategies in response to negative emotions [37]. Given the possible positive effects of maternal RF on children’s emotional development, exploring the mediators that explain how RF promotes children’s emotion regulation and identifying moderators that modulate the effects of RF are pivotal. 

### 1.2. The Mediating Role of Maternal Meta-Emotion Philosophy

*Meta-emotion philosophy* (MEP) is defined as an individual’s beliefs and responses towards their own emotions and those of others, which are shaped by their own emotional experiences [22,23]. For parents, it refers to their awareness and acceptance of specific emotions, as well as guidance towards their children’s emotional swings [38]. It offers insight into how parents’ own meta-emotions are connected to their parenting behaviors when responding to their children’s emotions [39]. 

Gottman described two types of MEP: emotion coaching and emotion dismissing [22,23]. Parents who espouse emotion coaching are aware of their own and their children’s emotions. They are willing to discuss emotions with their children, encourage their children to express their feelings with empathy, take emotional experiences as opportunities for bonding parent-child relationships, and tend to raise children who develop adaptive emotion regulation skills. In contrast, parents who are more emotionally dismissing in their approach consider negative emotions as harmful and unimportant, they discourage emotional expression and attempt to treat their children’s emotions by ignoring, minimizing, or punishing them [40], which tends to contribute to child emotion dysregulation and other maladaptive outcomes [22,41,42]. 

According to the MEP theory, parents’ beliefs and responses toward emotion guide their own emotional socialization and further affect their children’s emotional competence and social adjustment [43]. In support of these postulations, results from several studies have demonstrated that children whose parents were higher in emotion coaching showed less stress, greater physiological regulation, as well as better social adjustment, emotional competence, and general well-being [22,41,44]. Children whose mothers were higher in emotion coaching also demonstrated more secure parent-child attachment and better emotional regulation as compared to those with mothers higher in emotion dismissing [42]. 

From a theoretical perspective, dimensions of MEP can be considered as mediators in the relation between PRF and the emotional development of children. According to the social information processing model of parental reflective functioning [17], reflective functioning skills facilitate the cognitive processes with which mothers can interpret their own and their children’s intentions and past parent-child interactions. In this model, mothers with PRF skills begin the interpretive process by encoding and evaluating cues exhibited by themselves and their children in a specific context. After considering their own and their child’s internal experiences and mental states, they clarify goals in the following interactions and consider potential responses. While deciding on a response in the mother-child interaction, the social information processing circle begins again and mothers use reflective skills to continue to evaluate themselves and their child’s internal state [17]. In this regard, a high PRF would be expected to facilitate adaptive parental responses, which enables parents to show supportive emotion coaching practices, especially when parent-child dyads are placed in stressful emotional contexts [25]. In contrast, mothers with a low PRF may lack the ability to effectively process social information when reacting to their children’s negative emotions, become overly stressed themselves and thus adopt the emotion dismissing style.

### 1.3. The Moderating Role of Gender and Age

Given that both parents and children influence how parents react to children’s emotions, we further speculated that the relations between PRF and meta-emotion philosophies may vary as a function of a child’s gender and age. In terms of child gender, girls are more often socialized to be more attuned to emotions and relationships from an early age, whereas boys may be encouraged to suppress emotional expression and prioritize individual achievement [45]. When children are placed in negative emotional contexts, girls may be more likely to seek out emotional support from their caregivers, while boys may be more likely to engage in active coping strategies that involve problem-solving and goal-directed behaviors [46,47]. As well, when parents and children were asked to discuss past emotional experiences, parents referred to emotions with preschool girls more often than with boys in their discussion [48,49]. Given these associations, on a more exploratory basis, we considered the moderating role of gender in the relations between PRF and meta-emotion philosophies. 

In terms of child age, there is evidence suggesting that parents’ coaching of children’s negative emotions increased from ages 5 to 11 years, whereas awareness and acceptance of their children’s negative emotions decreased between 5 and 9 years [50]. As children get older, mothers may become less sensitive to their children’s negative emotions because parents tend to hold the belief that with increasing age, a child should be increasingly able to deal with distress independently [51]. Accordingly, we postulated that with the increasing child age, the associations between PRF and meta-emotion philosophies would be attenuated.

### 1.4. The Present Study

In the present study, we examined a complex conceptual model linking PRF, parental meta-emotion philosophies, and child emotion regulation among mothers of young Chinese children. First, this model postulates that emotion coaching and dismissing philosophies mediate the association between maternal PRF and children’s emotional regulation. Second, a child’s gender and age moderate the links between maternal PRF and emotion coaching and dismissing.

As an extension to existing studies mostly conducted in Western cultures, we examined these associations in a sample of mothers from mainland China. Compared to Western cultures, in China, parental authority, control, and harsh discipline are more strongly emphasized [52,53]. Chinese parents also tend to have higher expectations for their children, especially in terms of academic success [54], which may put pressure on both parents and children [55,56] and increase the difficulty of understanding their children’s mental states [57,58,59]. 

In addition, parents from different cultural groups may socialize their children’s emotions in diverse ways. For example, in comparison with White American parents, Korean immigrant parents demonstrated lower levels of awareness and acceptance towards their children’s negative emotions, and were less engaged in emotion coaching [60]. Chinese mothers were also found to use coaching strategies less frequently in response to their children’s emotional expressions compared with Italian mothers and were more likely to display punitive, dismissing, and distressed responses to their children’s emotional expressions [61]. Accordingly, the Chinese cultural background is an important and unique setting for exploring the links between PRF, meta-emotion philosophies, and child emotion regulation. 

We hypothesized that: (1) maternal PRF would be positively associated with child emotion regulation; (2) maternal emotion coaching and dismissing would mediate the relation between maternal PRF and child emotion regulation; and (3) child age would moderate the relation between maternal RF and emotion coaching or dismissing. No prior hypotheses were made with regard to potential child gender differences in the relation between PRF and emotion coaching or dismissing.

## 2. Method

### 2.1. Participants

The sample consisted of 667 mothers aged 25–47 years, with children attending one of three kindergartens in Shanghai, P.R. China. Table 1 shows the demographic characteristics of the parents and children. 

### 2.2. Procedure

Participants were recruited by graduate students through kindergartens in November 2021. Mothers were told they would participate in a study about social development in early childhood and provided informed consent for this study. There was no compensation provided to them. They were requested to complete a series of questionnaires online. Participation in this research was voluntary, and mothers were told that their personal information was kept confidential and they could stop participating at any time. The study was approved by the University Committee on Human Research Protection at ECNU. 

### 2.3. Measures

#### 2.3.1. Parental Reflective Functioning

Maternal reflective functioning was assessed using the Chinese version of the *Parental Reflective Functioning Questionnaire* (PRFQ) [62], a 12-item self-report questionnaire rated on a 7-point Likert scale (1 = *strongly disagree*; 7 = *strongly agree*). The PRFQ includes three subscales: (1) pre-mentalizing modes (PM), which refer to non-mentalizing modes, that is, parents are unable to grasp their children’s mental states (e.g., “The only time that I am sure about what my child wants is when he/she smiles at me”); (2) interest and curiosity in mental states (IC), which reflects the parents’ interest or curiosity about their children’s inner world (e.g., “I am often curious to find out how my child feels”); and (3) certainty about mental states (CMS), which shows that parents are certain about their children’s opaque mental states (e.g., “I always know what my child wants”). These three subscales reflect distinct features of PRF, reflecting the complexity and multi-dimensionality of PRF [62,63]. 

The Chinese version of the PRFQ has previously demonstrated good psychometric properties and construct validity (CFI = 0.929; TLI = 0.904; RMSEA = 0.065) [53]. In this study, Cronbach’s α = 0.0.69, 0.72, 0.79, and McDonald’s ω = 0.69 (95% CI [0.61, 0.74]); 0.72 (95% CI [0.64, 0.79]; 0.79 (95% CI [0.73, 0.82]), for the PM, IC, and CMS subscales, respectively.

#### 2.3.2. Meta-Emotion Philosophy

The *Parental Meta-Emotion Philosophy Scale* (PMEPS) was developed for use in China [64] based on previous work by Gottman [23]. The original version has four subscales, but only the emotion coaching and emotion dismissing subscales were of interest in this study. Emotion coaching reflects what extent parents are sensitive to their children’s and their own emotion, and guide their children’s emotional responses to different situations (15 items, e.g., “When my child is angry/sad, I go to hug or comfort him/her”). Emotion dismissing reflects what extent parents try to make their children ignore or reject negative emotional expressions, and do not help or encourage their children to deal with various situations (7 items, e.g., “I don’t allow my child to show angry/sad”). The PMEPS is rated on a Likert scale, ranging from 1 (*totally disagree*) to 6 (*totally agree*). The reliability indices of Cronbach’s α were 0.86 and 0.74, and McDonald’s ω were 0.87 (95% CI [0.84, 0.89]) and 0.76 (95% CI [0.72, 0.79]) for the emotion coaching and emotion dismissing subscales, respectively.

#### 2.3.3. Child Emotion Regulation

Mothers rated their children’s emotion regulation using the Chinese version of the *Emotion Regulation Checklist* (ERC) [65]. This scale was used to reflect their children’s emotion regulation levels evaluated by adults (including parents and teachers). The ERC comprises two scales, with items rated on a 4-point Likert scale (1 = *never*; 4 = *always*): (1) emotion regulation, which includes items that assess the children’s ability to alter their experience and expression of emotions (8 items, e.g., “Can say when she/he feels sad, angry or mad, fearful, or afraid”); and (2) emotional liability/negativity, which refers to mood liability, lack of flexibility, and negative affect dysregulation (16 items, e.g., “My child is easily frustrated”). The scale has displayed good reliability and validity in various cultural backgrounds [66,67]. As item 23 had poor loading in the revised scales in different cultures, it was removed in this study as well. In this study, the emotion regulation and emotional liability/negativity subscales showed good internal consistency, with Cronbach’s α = 0.70 and 0.78, and McDonald’s ω = 0.71(95% CI [0.65, 0.76]) and 0.79(95% CI [0.75, 0.83]). Scores of the two subscales were positively correlated (*r* = 0.36, *p* < 0.01) and averaged, with emotional liability/negativity reversely coded, to create an index for the children’s emotion regulation abilities. 

### 2.4. Data Analysis

Data were analyzed using SPSS 24 and PROCESS Marco 4.1 developed by Hayes (2013). First, we examined the descriptive statistics for all of the variables to ensure their normal distribution. Next, we examined Pearson’s correlations between the study variables, as well as their correlations with the participants’ demographic characteristics. We then tested the mediation role of emotion coaching and dismissing between PRF and child emotion regulation using PROCESS Model 4, and tested whether a child’s gender and age moderated the relations between PRF and MEPs using PROCESS Model 7. 

## 3. Results

### 3.1. Preliminary Analysis

Descriptive statistics of the study variables and their correlations are presented in Table 2. The three subscales of PRFQ were correlated with maternal meta-emotion philosophies: CMS and IC positively, and PM negatively related to emotion coaching; CMS and IC negatively, and PM positively related to emotion dismissing. PRFQ was significantly correlated with children’s emotion regulation: CMS and IC positively, and PM negatively related to emotion regulation. Finally, emotion coaching was positively correlated with the children’s emotion regulation, while emotion dismissing was negatively correlated with the children’s emotion regulation.

The correlations between social-demographic variables and the study variables (see Table 3) showed that: (1) child age was negatively correlated with IC and emotion coaching and positively correlated with emotion dismissing; (2) both the fathers’ and mothers’ higher educational levels and higher income were related with the mothers’ lower PM and higher IC; and (3) both parents’ educational and income levels were moderately negatively correlated with emotion dismissing. 

We also examined differences between mothers of boys and girls in their RF and meta-emotion philosophies, as well as gender differences in emotion regulation. The results showed that mothers of boys had significantly higher levels of PM than mothers of girls (*F* = 5.22, *p* < 0.05), and the emotion regulation of boys was significantly lower than that of girls (*F* = 8.92, *p* < 0.01), but there was no gender difference in the meta-emotion philosophies. 

### 3.2. The Mediating Role of Emotion Coaching and Dismissing 

Based on the PROCESS Model 4, emotion coaching and dismissing were tested for mediating effects between maternal PRF and the children’s ER, after controlling for the gender and age of the children, parental education, and income level. The effects of the three dimensions of PRF were examined in separate models. The results are displayed in Table 4 and Table 5. 

The direct predictive effect of PM on ER was significant (*β* = −0.46, *t* = −11.90, *p* < 0.001). When the mediating variables were added, the direct effect of PM on ER was still significant (*β* = −0.25, *t* = −5.78, *p* < 0.001). The indirect effect between PM and ER was significant (*β* = −0.21, 95% CI [−0.27, −0.15]). PM negatively predicted emotion coaching (*β* = −0.25, *t* = −6.06, *p* < 0.001) and positively predicted emotion dismissing (*β* = 0.52, *t* = 15.42, *p* < 0.001). Emotion coaching positively predicted children’s ER (*β* = 0.18, *t* = 4.68, *p* < 0.001), and emotion dismissing negatively predicted children’s ER (*β* = −0.31, *t* = −6.74, *p* < 0.001).

The direct effect of CMS on ER was significant (*β* = 0.25, *t* = 6.11, *p* < 0.001). When the mediating variables were added, the direct effect of CMS on ER was smaller but still significant (*β* = 0.09, *t* = 2.21, *p* < 0.05). The indirect effect between CMS and ER was significant (*β* = 0.16, 95% CI [0.10, 0.22]). CMS positively predicted emotion coaching (*β* = 0.48, *t* = 12.97; *p* < 0.001) and negatively predicted emotion dismissing (*β* = −0.19, *t* = −4.75, *p* < 0.001). Emotion coaching positively predicted the children’s ER (*β* = 0.16, *t* = 3.75, *p* < 0.01), and emotion dismissing negatively predicted the children’s ER (*β* = −0.44, *t* = −10.89, *p* < 0.001).

The direct effect of IC on ER was significant (*β* = 0.18, *t* = 4.14, *p* < 0.001). When mediating variables were added, the direct effect of IC on ER was no longer significant (*β* = 0.03, *t* = 0.76, *p >* 0.05). The indirect effect between IC and ER was significant (*β* = 0.15, 95% CI [0.08, 0.21]). IC positively predicted emotion coaching (*β* = 0.41, *t* = 10.69; *p* < 0.001) and negatively predicted emotion dismissing (*β* = −0.15, *t* = −3.61, *p* < 0.01). Emotion coaching positively predicted the children’s ER (*β* = 0.19, *t* = 4.60, *p* < 0.001), and emotion dismissing negatively predicted the children’s ER (*β* = −0.44, *t* = −11.02, *p* < 0.001).

### 3.3. The Moderation of Child Gender and Age between PRF and MEP

**Figure 1:**
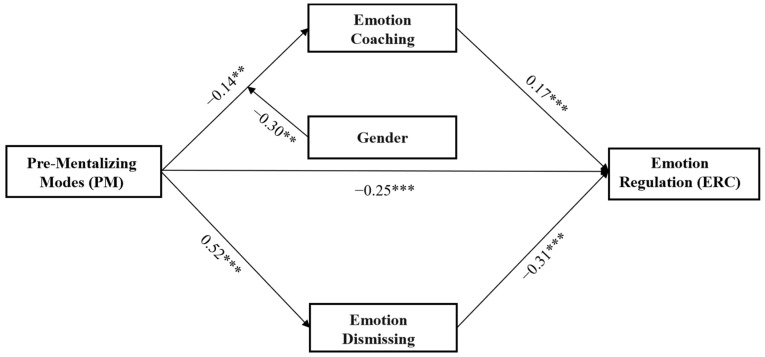
The model describes how gender moderated the relation between PM and emotion coaching, and how emotion coaching and dismissing mediated the relation between PM and children’s emotion regulation (** *p* < 0.01, *** *p* < 0.001).

**Figure 2:**
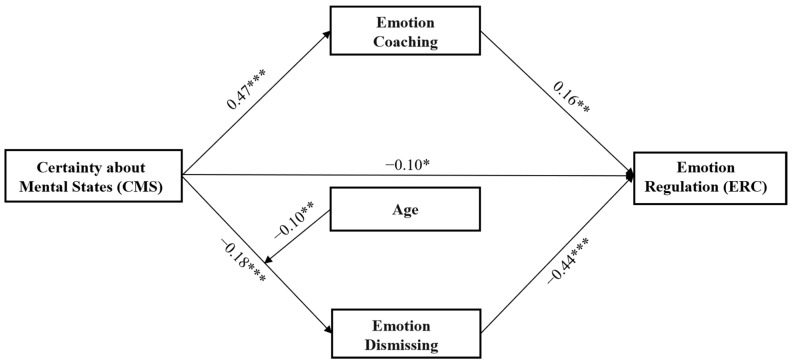
The model describes how age moderated the relation between CMS and emotion dismissing, and how emotion coaching and dismissing mediated the relation between CMS and children’s emotion regulation (* *p* < 0.05, ** *p* < 0.01, *** *p* < 0.001).

**Figure 3:**
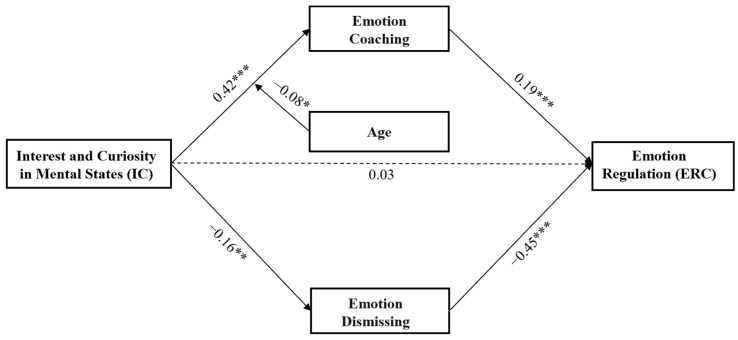
The model describes how child age moderated the relation between IC and emotion coaching, and how emotion coaching and dismissing mediated the relation between IC and children’s emotion regulation (* *p* < 0.05, ** *p* < 0.01, *** *p* < 0.001).

**Figure 4:**
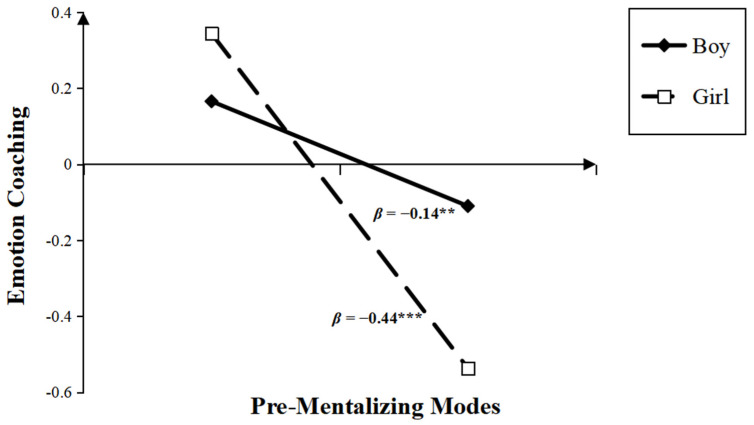
Interaction between child gender and PM on emotion coaching (** *p* < 0.01, *** *p* < 0.001).

Finally, we tested whether a child’s gender and age moderated the relations between PRF and MEPs using the PROCESS Model 7 (see Table 6 and Table 7, Figure 1, Figure 2 and Figure 3). After controlling for child age, parental education, and income level, the results showed that the interaction between gender and maternal PM was significant (*β* = −0.30, *t* = −3.71, *p* < 0.01), whereas the interactions between gender and the other two dimensions of PRF were not significant (for CMS, *β* = −0.02, *t* = −0.31, *p* > 0.05; for IC, *β* = 0.01, *t* = 0.08, *p* > 0.05). The simple slope test (see Figure 4) revealed that the negative effect of maternal PM on emotional coaching was stronger for girls (*β* = −0.44, *t* = −6.69, *p* < 0.001) than for boys (*β* = −0.14, *t* = −2.75, *p* < 0.01). 

**Figure 5:**
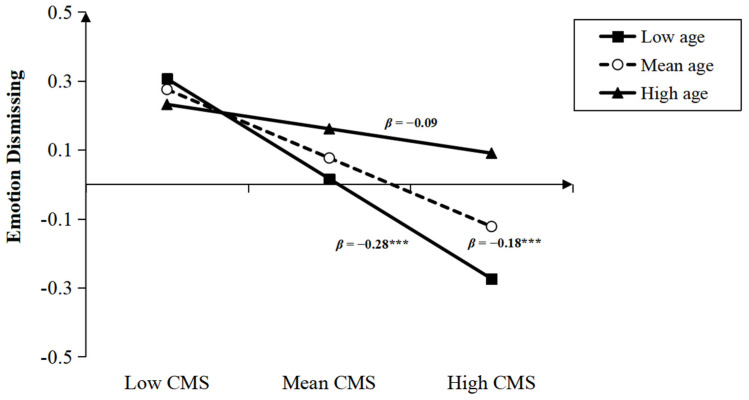
Interaction between child age and CMS on emotion dismissing (*** *p* < 0.001).

**Figure 6:**
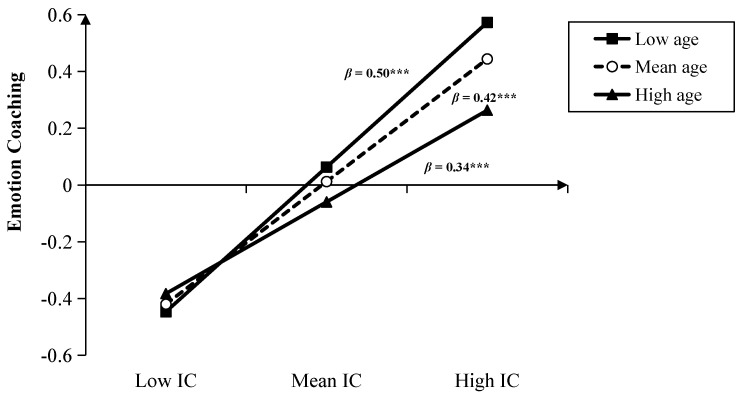
Interaction between age and IC on emotion coaching (*** *p* < 0.001).

After controlling for child gender, parental education, and income level, the result showed that the interaction between child age and maternal CMS was significant (*β* = −0.10, *t* = 2.47, *p* < 0.01). The simple slope test (see Figure 5) revealed that the negative effect of maternal CMS on emotional dismissing was stronger for younger children (i.e., child age was 1 SD below the mean. *β* = −0.28, *t* = −5.15, *p* < 0.001) and non-significant for older children (i.e., child age was 1 SD above the mean. *β* = −0.09, *t* = −1.05, *p* > 0.05). The interaction between child age and maternal IC was also significant (*β* = −0.08, *t* = −2.16, *p* < 0.05). The simple slope test (see Figure 6) revealed that the positive association between maternal IC and emotional coaching was stronger for younger children (*β* = 0.50, *t* = 8.94, *p* < 0.001) than for older children (*β* = 0.34, *t* = 6.31, *p* < 0.001). Nevertheless, the interactions between age and PM were not significant (*β* = −0.04, *t* = −1.11, *p* > 0.05).

## 4. Discussion

In the present study, we examined whether Chinese mothers’ meta-emotion philosophies (MEP) of emotion coaching and dismissing mediated the association between maternal parenting reflective practice (PRF) and children’s emotion regulation (ER), as well as the moderating effect of children’s gender and age on the associations between PRF and MEPs. The results revealed significant mediating and moderating effects for the three dimensions of PRF. Specifically, higher levels of interest and curiosity (IC) predicted higher levels of emotion coaching and lower levels of emotion dismissing, which were both associated with children’s ER. The positive association between IC and emotional coaching was stronger for younger children. Maternal certainty about mental states (CMS) was directly and indirectly (via MEPs) related to the children’s emotion regulation, and the negative association between CMS and emotional dismissing was also stronger for younger children. In contrast, maternal pre-mentalizing modes (PM) were negatively associated with the children’s ER, both directly and indirectly through emotional coaching and dismissing, and the negative effect of PM on emotional coaching was stronger for girls than boys.

### 4.1. Mediating Role of Meta-Emotion Philosophies

The three dimensions of PRF assessed in the present study were all associated with the emotion regulation of children directly and indirectly via the mediated pathways of emotion coaching and dismissing. These findings are consistent with previous studies, indicating that maternal emotion coaching can reduce children’s internalized and externalized problems and promote their social adaptation and emotional development [44,68]. In contrast, maternal emotion dismissing negatively predicted children’s emotion recognition [44] and emotion regulation [69]. According to the social biofeedback theory of parental effect mirroring [70], children are not born with the ability to internalize emotions as mental states. Instead, children learn to categorize, represent, and control their inner states through affect-regulation interactions with their caregivers. Sensitive parents tend to attune their own affective responses to modulate their children’s emotional states, and children can learn that by externalizing their internal emotional states, they can achieve successful regulation of their affective impulses [70,71]. Mothers who have an emotion-coaching strategy tend to accept their children’s emotions and use verbal coaching to help their children understand and cope with their own emotional problems appropriately [22,23], and their children will learn to regulate their emotions by imitating their parents’ emotion-coping. 

According to Fonagy’s theory [6], mothers with a better PRF may not immerse themselves in their own emotional experiences and, instead, they reflect on the child’s emotional experience and guide more positive social-emotional interaction with their children when their children are in stressful contexts [8]. Our findings showed that mothers with more interest and curiosity about their children’s mental states were more likely to show emotional coaching and less likely to show emotion dismissing. In turn, a mother’s emotional coaching and dismissing predicted their children’s emotion regulation skills. Previous studies have found that lower levels of interest and curiosity in a child’s mental states were associated with less emotional awareness of the parents’ own distress and less tolerance of infant distress [72,73]. It can be inferred that mothers who were unaware of their own emotions may have decreased awareness of their child’s emotions, and it may further lead to negative beliefs and coping strategies in response to their children’s negative emotions [74]. This finding is also consistent with the previous studies, showing that maternal interest and curiosity were associated with the infants’ secure attachment [62] and that infants who had a secure attachment with their caregivers were more likely to develop better emotional regulation skills as they grew older [57,75,76].

Conversely, pre-mentalizing modes were both directly and indirectly associated with the children’s ER, suggesting that the parents’ inability to grasp their children’s mental states may affect both maternal MEPs and the children’s emotional adjustment. This result was consistent with the findings of other studies, indicating that pre-mentalizing modes were associated with the mothers’ less tolerance of stress [9,77] and reduced sensitivity and warmth [33,53]. Mothers with higher levels of pre-mentalizing modes may react to their child’s behaviors in a more impulsive and dismissing way. Emotionally dismissing mothers tend to view negative emotions as toxic or overwhelming, invalidate or criticize their children’s emotions, and want to avoid or protect their child or themselves from negative emotions [78], rather than take the time to consider what their child might be feeling or thinking. Pre-mentalizing modes may interfere with mothers’ ability to understand their children’s mental states, leading to less supportive and empathetic responses to children, impacting the way they treat their children’s negative emotions, and ultimately affecting the child’s emotional and social development. 

Additionally, maternal certainty about mental states also positively predicted child emotion regulation, with MEPs partially mediating the relation between them. Other research also found positive associations between certainty about mental states and positive parenting practices [30], as well as negative associations between certainty about mental states and child internalizing and externalizing problems [63]. However, according to Luyten [62], too high or too low scores on this subscale may reflect overly certain (*hypermentalizing*, a lack of recognition of the opacity of mental states) or overly uncertain (*hypomentalizing*, a lack of certainty about the mind) about the mental states of the child and their own. Certainty about mental states and parenting practice seemed to have a non-linear association. An overly certain or uncertain perception of mental states may have negative implications for parenting behaviors and the social-emotional development of children. As Rostad and Whitaker [30] argued, parents may overestimate their knowledge about their children’s mental states in order to feel better as parents or caregivers are more certain as a result of an increased awareness of their children’s needs. It is also possible that if parents are too uncertain and unclear about their child’s mental state, they may struggle to accurately understand their children’s needs or even dismiss the existence of their inner world. When a mother is unable to correctly identify her child’s mental state, due to this ambiguity, she may display withdrawal behaviors, which can also increase the risk of social-emotional difficulties in her child [63,79]. In sum, the associations between certainty about mental states, parenting practices, and the social-emotional development of children need to be further explored in the future.

### 4.2. Moderating Effects of Child Age and Gender

In this study, mothers of older children exhibited higher levels of pre-mentalizing modes. This finding was consistent with previous research indicating that parents of 8–10 years old children exhibited more pre-mentalizing modes than parents of 3–5 years old children [80]. Our results indicate that even within the preschool period, there were differences in the mothers’ ability to interpret their children’s inner world based on the children’s age. It has been argued that as children develop, emotional regulation strategies become more complex and diverse, which may both facilitate and hinder the mothers’ understanding of their children’s mental states [77,80]. 

Our findings also indicated that mothers of younger children with higher levels of interest/curiosity and certainty about mental states exhibited more emotion coaching and less emotion dismissing than mothers of older children. This suggests that as children grow, mothers may reduce their guidance and assistance in response to their children’s emotional disturbances. This result is in line with previous studies. For example, as children develop, they regulate emotional behaviors more maturely [81], and compared with older children, younger children tend to receive more acceptance and support for emotional expression [82]. In sum, mothers may believe that as children get older, they need to learn to manage their emotions more independently. Thus, compared with mothers of younger children, mothers of older children may show more emotional dismissing and less emotional coaching, which is less contingent on the mothers’ PRF. 

Before discussing the moderating role of a child’s gender in the links between their mothers’ reflective functioning and meta-emotion philosophies, we must first address the differences between mothers of boys and girls with regard to pre-mentalizing modes. We found that mothers of boys had significantly higher levels of pre-mentalizing modes than mothers of girls. This finding was inconsistent with a previous study conducted in Italy, which found that parents of girls had higher scores on pre-mentalizing modes than parents of boys [80]. Of note, this study included both mothers and fathers of children aged 3–10 years. According to Fonagy [83], parents’ capacity to engage in proper mentalizing is influenced not just by their own PRF but also by their child’s characteristics. Given this theoretical basis, the inconsistency may be explained by the different age groups of the samples. Specifically, in the preschool period, girls tend to display less externalizing emotions, such as anger, and more positive emotions because of biologically-based advantages in language and self-regulation abilities [45]. In contrast, in later childhood and adolescence, as gender role expectations change, boys are socialized to suppress or hide their emotions, and girls begin to express more externalizing emotions, such as anger, to their parents [45], which may cause parents to make more maladaptive and malevolent attributions about girls’ inner states. Given the inconsistent results, more studies are needed to investigate how child characteristics and parental gender affect PRF.

Regarding the moderating effect of child gender, our findings indicated that the negative effect of maternal pre-mentalizing modes on emotional coaching was stronger for mothers of girls than boys. Most previous studies have not shown child gender differences in the relation between maternal mentalization and parenting behaviors, but some research suggested that a mother-daughter relationship is more likely to develop a trajectory of reciprocal responsiveness than a mother-son relationship, because mothers are prone to create more intimate relations with daughters [84]. Feldman [85] also found that negative emotions, especially expressions of anger, may be more damaging for the mother-daughter than the mother-son relationship synchrony. According to Radke-Yarrow and Kochanska, mothers are more accepting of negative emotional expressions (e.g., anger and frustration) from boys than girls, and attend to boys’ anger with concern and ignore girls’ anger [86,87]. In traditional Chinese culture, daughters are emotionally intertwined with family and bear burdensome emotional expectations more than sons [88]. Hence, it can be inferred that when mothers lack mature mentalization, they may give less guidance to girls than boys when they face the challenges of negative emotions.

### 4.3. Limitations and Future Directions

The current study revealed that mothers’ emotion coaching and dismissing mediated the effect of maternal PRF on their children’s ER, and a child’s gender and age moderated the relation between the three dimensions of PRF and MEPs of mothers. However, some limitations of the study should be considered when interpreting the results, with an eye toward future research. First, the sample was relatively homogeneous, comprising mainly mothers from Shanghai, a metropolitan area in P.R. China. Parents and children from other cities in China with lower economic levels and from other countries and ethnicities may exhibit different levels of PRF and emotional socialization behaviors. High-risk families from clinical samples should also be included in future investigations. 

Second, only maternal self-report scales were employed to access PRF, emotion coaching and dismissing, and the children’s ER. As such, shared method variance may have heightened associations between the variables. Future research should replicate these results using multi-source assessments, including teacher reports, child self-reports, and narrative interviews and observations. For example, future research may use the *Parental Development Interview* (PDI) [8] to ask parents to describe their emotional experiences of parenting, the *Emotion Discussion Task* [89] to observe maternal emotion coaching and dismissing in a mother-child discussion about emotion-eliciting events [38], and the *Disappointing Gift Task* to observe the children’s reaction to the gift and facial expression of emotion [90]. 

Third, this study was cross-sectional in design. Longitudinal studies that incorporate repeated measures over time are necessary to understand the complex and possibly bi-directional dynamics of a parent-child relationship. Finally, only mothers participated in this study. Father-child dyads or triadic interactions should be considered. Future research is needed to investigate whether the parents’ gender and SES could affect the relations between PRF and their children’s outcomes.

Fourth, this study was conducted during the COVID-19 pandemic, although the widespread lockdown had been lifted when the research began. The stress of the COVID-19 pandemic was found to intensify the psychological problems among children, leading to disruptions in their daily routines and an increase in family conflict [91]. The economic, social, or psychological factors related to the pandemic could have influenced parental behaviors or attitudes, potentially introducing confounding variables or affecting the validity of the results. Given the unique circumstances of the pandemic, caution should be exercised when generalizing our findings.

In conclusion, our findings suggest that the emotional regulation of children can be predicted by maternal PRF via mediated pathways through emotion coaching and dismissing. Therefore, it is necessary to improve PRF in order to promote the better social-emotional development of children and pay special attention to how a child’s characteristics may affect their parents’ mentalization.

## Figures and Tables

**Table 1:** Descriptive characteristics of the sample.

Parental Characteristics	Mothers	Fathers
Age-*M* (*SD*)	35.16 (3.66)	36.91 (4.35)
Educational level (%)		
Primary education or below	0.2	0.2
High school education	9.7	7.7
College degree	21.1	17.2
Bachelor’s degree	47.1	47.6
Master’s degree or above	21.9	27.4
Income level (%)		
<2000 CNY	6	1.4
2000–5000 CNY	8.7	4.5
5000–10,000 CNY	30.9	18.3
10,000–15,000 CNY	21.9	16.6
15,000–20,000 CNY	14.5	16.9
>20,000 CNY	17.9	42.4
Employment status (%)		
In employment	81.4	84.3
Unemployment	6.3	0.3
Freelance work	3.8	4.4
Others	8.5	11.0
Children characteristics		
Age-*M* (*SD*)	4.97(0.97)	
Sex		
Male (*n*) %	356 (53.4)	
Female (*n*) %	311 (46.6)	
Siblings		
Only one child (*n*) %	434 (65.1)	
2 kids (*n*) %	226 (33.9)	
3 kids (*n*) %	7 (1.0)	

**Table 2:** Correlations between maternal RF, emotion coaching and dismissing, and child emotion regulation.

	1	2	3	4	5	6	7
1 PM	1						
2 CMS	−0.18 **	1					
3 IC	−0.10 *	0.37 **	1				
4 Emotion Coaching	−0.25 **	0.49 **	0.47 **	1			
5 Emotion Dismissing	0.59 **	−0.18 **	−0.22 **	−0.27 **	1		
6 Liability/Negative Emotion	0.43 **	−0.17 **	−0.14 **	−0.19 **	0.42 **	1	
7 Emotion Regulation	−0.28 **	0.24 **	0.20 **	0.40 **	−0.34 **	−0.36 **	1
*M*	3.00	5.11	6.08	4.65	2.82	1.98	3.22
*SD*	1.14	1.01	0.82	0.58	0.82	0.34	0.40

PM: Pre-Mentalizing Modes: CMS: Certainty about Mental States: IC: Interest and Curiosity in Mental States. * *p* < 0.05, ** *p* < 0.01

**Table 3:** Correlations between social-demographic variables and the study variables.

	PM	CMS	IC	Emotion Coaching	Emotion Dismissing	Emotion Regulation
Child Age	0.06	−0.02	−0.08 *	−0.10 *	0.10 *	−0.002
Mother Educational level	−0.20 ***	0.04	0.16 ***	0.08 *	−0.31 **	0.07 ^†^
Father Educational level	−0.18 ***	0.07 ^†^	0.10 **	0.10 *	−0.29 **	0.09 *
Mother Income level	−0.16 ***	0.06	0.17 ***	0.08 †	−0.25 **	0.07 ^†^
Father Income level	−0.20 ***	0.11 **	0.13 **	0.07	−0.25 **	0.04

^†^: 0.05 ≤ *p* < 0.1, * *p* < 0.05, ** *p* < 0.01, *** *p* < 0.001.

**Table 4:** Standardized direct, indirect, and total effects of the parallel mediating models.

Independent Variables	Direct Effect (95% CI)	Indirect Effect (95% CI)	Total Effect (95% CI)
PM	−0.25 (−0.34, −0.17)	−0.21 (−0.27, −0.15)	−0.46 (−0.54, −0.38)
CMS	0.09 (0.01, 0.18)	0.16 (0.10, 0.22)	0.25 (0.17, 0.34)
IC	0.03 (−0.05, 0.11)	0.15 (0.08, 0.21)	0.18 (0.09, 0.26)

**Table 5:** The mediating role of maternal emotion coaching and dismissing in the relation between maternal RF and children’s ER.

	Outcomes	Predictors	Fit Indexes		Significance of Regression Coefficients	
			*F*	*R* ^2^	*β*	*t*
Model 1	Emotion coaching	PM	6.89 ***	0.08	−0.25	−6.06 ***
	Emotion dismissing	PM	49.47 ***	0.39	0.52	15.42 ***
	Children’s ER	PM	29.12 ***	0.32	−0.25	−5.78 ***
		Emotion coaching			0.18	4.68 ***
		Emotion dismissing			−0.31	−6.74 ***
Model 2	Emotion coaching	CMS	26.04 ***	0.25	0.48	12.97 ***
	Emotion dismissing	CMS	14.48 ***	0.16	−0.19	−4.75 ***
	Children’s ER	CMS	24.71 ***	0.54	0.09	2.21 *
		Emotion coaching			0.16	3.75 **
		Emotion dismissing			−0.44	−10.89 ***
Model 3	Emotion coaching	IC	18.19 ***	0.19	0.41	10.69 ***
	Emotion dismissing	IC	12.93 ***	0.14	−0.15	−3.61 **
	Children’s ER	IC	24.04 ***	0.28	0.03	0.76
		Emotion coaching			0.19	4.60 ***
		Emotion dismissing			−0.44	−11.02 ***

* *p* < 0.05, ** *p* < 0.01, *** *p* < 0.0013

**Table 6:** The moderating effect of gender on the relation between PM and emotion coaching/dismissing.

Outcomes	Predictors	Fit Indexes		Significance of Regression Coefficients	
		*F*	*R^2^*	*β*	*t*
Emotion coaching	PM	7.89 ***	0.10	−0.14	−2.75 **
	gender			−0.12	−1.58
	PM × gender			−0.30	−3.71 **
Emotion dismissing	PM	43.21 ***	0.39	0.52	12.32 ***
	gender			0.00	0.01
	PM × gender			−0.01	0.17
Children’s ER	PM	31.54 ***	0.32	−0.26	−6.03 ***
	Emotion coaching			0.17	4.50 ***
	Emotion dismissing			−0.31	−6.74 ***

** *p* < 0.01, *** *p* < 0.001.

**Table 7:** The moderating effect of child age on the relation between CMS/IC and emotion coaching/dismissing.

Outcomes	Predictors	Fit Indexes		Significance of Regression Coefficients	
		*F*	*R^2^*	*β*	*t*
Emotion coaching	CMS	22.93 ***	0.25	0.47	12.93 ***
	age			−0.08	−2.11 *
	CMS × age			−0.04	−1.05
Emotion dismissing	CMS	13.56 ***	0.17	−0.18	−4.70 ***
	age			0.06	1.67
	CMS × age			−0.10	2.47 **
Children’s ER	CMS	27.65 ***	0.29	−0.10	2.27 *
	Emotion coaching			0.16	3.68 **
	Emotion dismissing			−0.44	−10.85 ***
Emotion coaching	IC	16.61 ***	0.20	0.42	10.85 ***
	Age			−0.05	−1.46
	IC × age			−0.08	−2.16 *
Emotion dismissing	IC	11.68 ***	0.15	−0.16	−3.72 **
	age			0.05	1.40
	IC × age			0.06	1.62
Children’s ER	IC	26.85 ***	0.28	0.03	0.76
	Emotion coaching			0.19	4.54 ***
	Emotion dismissing			−0.45	−10.97 ***

* *p* < 0.05, ** *p* < 0.01, *** *p* < 0.001.

## Data Availability

Data is unavailable due to the ethical restrictions of the University Committee on Human Research Protection of ECNU.
